# Beyond symbolic algebra with quantum picturalism

**DOI:** 10.3389/fcogn.2026.1790789

**Published:** 2026-06-24

**Authors:** Selma Dündar-Coecke, Lia Yeh, Emmanuel Pothos, Muhammad Hamza Waseem, Bob Coecke

**Affiliations:** 1 Neurossance, Oxford, United Kingdom; 2 Centre for Educational Neuroscience, King's College London, London, United Kingdom; 3 Department of Computer Science, University of Oxford, Oxford, United Kingdom; 4 Department of Computer Science and Technology, University of Cambridge, Cambridge, United Kingdom; 5 Department of Psychology, City University, London, United Kingdom; 6 Clarendon Laboratory, Department of Physics, University of Oxford, Oxford, United Kingdom; 7 Perimeter Institute, Waterloo, ON, Canada; 8 Wolfson College, University of Oxford, Oxford, United Kingdom

**Keywords:** cognition, diagrammatic formalisms, education, epistemology, mathematics, Quantum Picturalism, quantum education, quantum theory

## Abstract

The more advanced the symbolic mathematics, the more impenetrable the meaning. This is particularly evident in quantum theory, where symbolic formalisms foreground states and objects. An alternative diagrammatic approach, Quantum Picturalism (QPic), privileges structure, relation, and transformation. As a category theoretic formalism, it supports reasoning through the composition of visually represented quantum processes, a distinction that not only concerns representation but also shapes how quantum phenomena are conceptualized, opening new educational possibilities and ways of knowing. The choice of formalism—algebraic or diagrammatic—is not neutral, but embodies underlying ontological commitments that shape which structural features of quantum phenomena—such as states vs. relations; objects vs. transformations—are made accessible. This paper examines the epistemological, cognitive, and educational significance of non-symbolic thought in mathematics, and in quantum mechanics in particular, using QPic as a guiding example. We raise a central question: can quantum processes be represented in ways that align more naturally with human cognition? What is new here is showing how the epistemological and cognitive aspects of non-symbolic formalism are brought into pedagogical practice. Such paradigm largely benefits from the *language of thought* hypothesis alongside theories of embodied cognition, dual coding, and conceptual metaphor theory to articulate a holistic framework in which multiple, interacting conceptual systems underpin understanding. Drawing on historical precedents—from Euclidean geometry to Leibniz's universal calculus of thought—we contend that human intuition works more naturally within relational and visual frameworks, particularly those that help complex ideas to be seen and manipulated as structured wholes. The broader impact of this paradigm lies in democratizing access to quantum innovation and rethinking what it means to *understand* the quantum world, offering an inclusive entry point into it.

## 1 Introduction

Quantum theory is one of the most sophisticated scientific theories ever invented, and the bedrock of many key technological advances in modern civilization. Yet it remains one of the most conceptually profound and pedagogically inaccessible areas of modern science. This difficulty stems largely from traditional approaches grounded in abstract algebraic formalism, which offer precision but limited cognitive transparency. As a result, access to quantum theory is often restricted, creating barriers that exclude many potential learners and constrain deeper understanding and creative thinking.

This is not just a pedagogical shortcoming but a representational one. The formalisms through which we reason are not neutral; they shape how we compute, and more crucially, what—and when—we are able to conceive. When structure remains implicit, symbolic fluency can be achieved without corresponding conceptual access.

The challenge is particularly consequential in the context of the emerging second quantum revolution—one that goes far beyond enabling innovations like MRI, lasers, navigation systems, or transistors. Today, quantum technologies are poised to transform computation, sensing, communication, artificial intelligence (AI), bioengineering, semiconductors, future telecoms and much more. Given the scale of these developments, the challenge is both to broaden access to quantum knowledge and also to make its underlying structures cognitively accessible.

One key obstacle to unlocking the real-world potential of this powerful, theory-driven field—especially at a scale that could shape society and culture—is a simple fact: quantum theory is a very difficult study. Traditional approaches to teaching the foundations of this theory involve complex vector spaces (Hilbert spaces), matrix algebra, differential equations, complex numbers, probability theory, and so on.

These mathematical requirements create significant barriers for young students precisely at the stage when key educational and career decisions are made. In England, Wales, and Northern Ireland, matrices are taught only within Further Mathematics, a subject taken by just 4.7% of A-level students in state schools in 2021 (The Office of Qualifications and Examinations Regulation, 2016; Mau, 2023). In Scotland, matrices appear only in Advanced Higher Mathematics, a qualification taken by more than ten times fewer students than National 5 Mathematics (Scottish Qualifications Authority, 2019, 2024). National examination reports further indicate that students taking Advanced Higher Mathematics perform particularly poorly on matrix multiplication (Scottish Qualifications Authority, 2023).

Taken together, these figures show how limited access to advanced mathematics significantly restricts early engagement with quantum theory and QIST topics, reinforcing structural barriers long before students encounter quantum content at university level (Dündar-Coecke et al., 2025). Beyond its theoretical complexity, practical engagement with quantum field requires a high-level of technical expertise. This includes proficiency in programming, the ability to work with specialized tolls and computational frameworks, and the use of optimization tools developed within advanced research and industrial environments—skills that often concentrated within commercial companies and specialized institutions.

While universities have struggled to keep pace with this rapidly evolving quantum industry, teaching quantum mechanics is typically reserved for physics undergraduates; even final year electives usually offer little more than a basic introduction. Most quantum computing experts, for example, usually have at least an MSc in quantum computing and/or a PhD in the topic or related ones (such as quantum foundations). All these prerequisites are an extremely narrow funnel; additionally, the particular set of hoops to make it to a quantum computing career comes with several biases, which narrow down the available human potential for such careers. In line with this, traditional approaches to teaching quantum theory often cater to mathematically gifted students, following what could be described as a “cognitive elitist” path.

**Figure 1:**
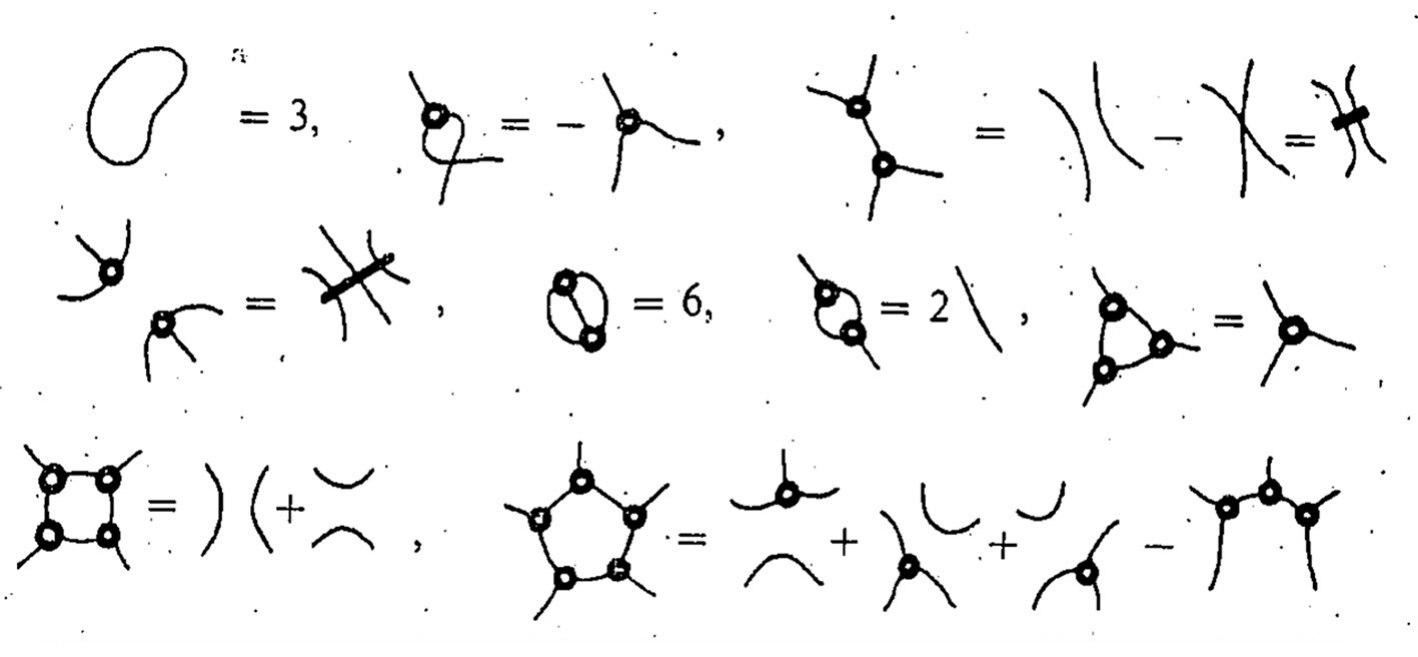
Roger Penrose was awarded the 2020 Nobel Prize in Physics for “propos[ing] critical mathematical tools to describe black holes” (Prize, 2020). These diagrams are from his paper in 1971 which introduced his now celebrated Penrose graphical notation—also known as tensor diagram notation—for reasoning about spins, i.e., angular momenta (Penrose, 1971).

There is, however, an alternative approach which provides a visual and intuitive framework, alleviating many of the traditional mathematical hurdles (especially multi-linear algebra) in physics education. Roger Penrose developed a diagrammatic calculus for multi-linear algebra (tensors), depicted in Figure 1. What Penrose did to revolutionize computing physical processes involving spin, has inspired a fully comprehensive and complete formalism for physical processes involving quantum systems that is now called Quantum Picturalism (QPic) (Coecke, 2006, 2010; Abramsky and Coecke, 2009). It is used as a tool to streamline quantum operations into intuitive visual representations.

At the heart of QPic's framework lies the ZX calculus, introduced by Coecke and Duncan (2008, 2011), which provides a rigorous yet intuitive approach to teaching and research through diagrammatic reasoning for quantum computation. Recent work has moreover achieved embedding of Penrose's spin diagrams into the ZX-calculus for all finite-dimensional quantum systems, elevating the applications of diagrammatic calculi to beyond that of purely qubit-based or purely spin-based systems (Wang et al., 2025).

Since then, the ZX calculus has proven highly versatile and has been widely adopted in both academic research and industry, with major quantum computing companies—such as Google, PsiQuantum, IBM, and Quantinuum—actively using it to design, optimize, and verify quantum algorithms. Its applications are diverse and impactful, particularly in the key industry use cases summarized in Table 1.

**Table 1:** Applications and use cases of ZX across fields and industries.

■Quantum compilation and optimization	■Quantum natural language processing and Artificial intelligence
ZX diagrams allow researchers to simplify and restructure quantum circuits visually, reducing gate counts and improving computational efficiency without resorting to complex algebraic manipulations (Coecke and Kissinger, 2017; Kissinger and van de Wetering, 2019a; de Beaudrap et al., 2020; Kissinger and van de Wetering, 2025), compile for more expressive quantum operations such as scalable notations (Carette et al., 2019), higher-dimensional quantum systems Yeh (2026), continuous-variable quantum computation (Shaikh et al., 2024), and dynamic protocols (de Felice et al., 2026).	Diagrammatic representations enable the mapping of linguistic structures onto quantum circuits, supporting quantum-assisted language understanding. Recent work demonstrates QPic's applicability to quantum-enabled explainable AI, where diagrammatic structures help translate complex quantum computations into forms interpretable by humans, bridging theory and practical applications (Coecke et al., 2020; Lorenz et al., 2023; Laakkonen et al., 2024; Duneau et al., 2024; Tull et al., 2024).
**■Measurement-based quantum computing**	**■Quantum simulation**
The calculus makes explicit the structural relationships between entanglement, measurement, and classical communication, streamlining the design and analysis of measurement-driven computation protocols (Coecke and Duncan, 2008; Duncan and Perdrix, 2009; Kissinger and van de Wetering, 2019b; Backens et al., 2021; McElvanney and Backens, 2023).	Complex simulations of quantum chemistry and physics systems can be expressed in the ZX-calculus (Shaikh et al., 2023), capturing interactions between qubits and bosons (de Felice et al., 2023), fermions McDowall-Rose et al. (2025), and spins (East et al., 2022; Wang et al., 2025).
**■Quantum foundations**	**■Quantum error correction**
ZX diagrams provide a visually intuitive framework for exploring fundamental quantum phenomena such as contextuality and non-locality, offering conceptual insights that complement formal and algebraic proofs (Coecke and Kissinger, 2017; Backens and Nabi Duman, 2015; Coecke et al., 2011; Pinzani et al., 2019).	By representing error syndromes and correction protocols diagrammatically, ZX calculus enables more intuitive design, analysis, and verification of fault-tolerant quantum circuits (de Beaudrap and Horsman, 2020; Gidney and Fowler, 2019; Huang et al., 2023; Townsend-Teague et al., 2023; Bombin et al., 2024; Rodatz et al., 2024).

Beyond quantum-specific applications, the category-theoretic foundations share structural similarities with graphical methods in other fields, including linguistics (Coecke, 2020; Coecke et al., 2010; Sadrzadeh, 2017), computer science (Coecke et al., 2013), machine learning (Gavranović, 2024; Koziell-Pipe et al., 2024), electronics (Boisseau and Sobociński, 2021), game theory (Ghani et al., 2018), and control theory (Bonchi et al., 2021).

While the ZX-calculus has demonstrated wide-ranging applications across diverse domains, its potential as a pedagogical and cognitive scaffold was first systematically examined in a 2023 pilot study, investigating whether quantum theory could fall within the zone of proximal development (ZPD) of high-school students when supported by QPic. The study involved 54 UK state-school students, randomly selected from a pool of 734 applicants, who completed an 8-week online program comprising 2-h weekly sessions delivered by seven qualified tutors under the supervision of three professors from the University of Oxford and Quantinuum. The course was designed to test whether QPic could lower the technical threshold typically associated with formal quantum education, enabling learners to both comprehend and apply core quantum concepts in novel problem-solving contexts, and closely mirrored the Picturing Quantum Processes module taught to undergraduates in the University of Oxford's Department of Computer Science, ensuring content fidelity and conceptual equivalence. Instruction combined guided visual reasoning with structured assessments to measure conceptual understanding and cognitive transfer. Outcomes exceeded expectations: 82% of participants passed the final assessment and 48% achieved distinction-level performance, while post-course surveys revealed increased motivation to pursue further study or careers in QIST. Together, these results constitute the first rigorous empirical evidence from a controlled, multi-tutor experimental framework demonstrating the efficacy of a diagrammatic pedagogy for quantum learning, with full methodological details reported in (Dündar-Coecke et al., 2025).

Moving forward, the objective of this paper is to provide cross-disciplinary insights into how this pictorial formalisms can bridge physics, computation, logic, linguistics, and cognitive science. This has promising sociocultural implications in demonstrating that reasoning patterns in one domain can inform and enrich approaches in others. This work is intended for audiences across three perspectives: philosophy of physics and mathematics (Section 2), cognitive science (Section 3), and science education (Section 4). From here onwards, the paper is organized into sections from the viewpoint of each of these three disciplines respectively, followed by concluding with discussion in Section 5.

## 2 The epistemological value of diagrammatic mathematics for learning quantum

Traditionally, mathematics—encompassing domains such as arithmetic, algebra, and geometry—has been practiced within an analytical paradigm that privileges abstraction, symbolic manipulation, and decomposition. Within this framework, complex systems are typically approached by breaking them into independent parts and reconstructing their behavior through formal rules and laws. This approach has proven remarkably effective for modeling many physical phenomena, particularly where systems can be treated as collections of well-defined components.

However, this framework becomes less straightforward when applied to systems whose behavior is fundamentally relational. In such cases, the properties of the whole do not arise from the parts in isolation, but from the structure of their interactions. A familiar example is weather systems; they cannot be understood by analyzing isolated variables such as temperature or pressure independently; rather, large-scale patterns emerge from the complex interactions between atmospheric components, making relational structure central to explanation. Quantum systems present this challenge in a more fundamental form because their behavior is inherently relational and resists reduction to independently specified parts. In such cases, the meaning of a system's state depends on its interactions and context, rather than being fully specified in isolation.

Although such fundamentally relational systems can be observed in several contexts, our cognition has been shaped through everyday interaction with a largely classical physical world, so it naturally rebels against the counterintuitive features of quantum theory. It is therefore natural to ask; how do humans, as cognitive agents, come to learn and reason about non-classical world, and how do we acquire the mathematical frameworks developed to describe it?

Several epistemological frameworks from the philosophy of science offer insights into this question. Among them, Popper's tripartite ontology of knowledge (Popper, 1972) and Hilbert's formalism are particularly relevant—not as competing accounts of physical reality, but as lenses for examining how mathematical knowledge is represented and structured.

Popper's tripartite ontology of knowledge (Popper, 1972) provides a useful lens for understanding how mathematical knowledge operates across different domains of reality: World 1 encompasses the physical realm—the tangible environment in which human beings exist, including neural systems, written symbols, and instruments. World 2 refers to the inner domain of subjective experience—perception, imagination, intuition, reasoning, and emotion. World 3, by contrast, consists of the products of thought that have achieved a form of objectivity: language, theories, artistic works, mathematics. In this view, the process of learning mathematics is a dynamic interplay among these three worlds. Human cognition (World 2) engages with mathematical artifacts (World 3) through reasoning, symbolic manipulation, and imagination, while being continuously grounded in the material and social realities of World 1—the physical act of writing equations, the use of visual aids, and participation in educational or cultural contexts.

Popper's framework assigns mathematics an objective yet human-centered status, and holds contrasting view to Platonist accounts, which treat mathematical entities as existing independently of the human mind in an abstract, non-physical realm. On the Platonist view, defended by proponents such as Kurt Gödel (Gödel, 1947), mathematical knowledge consists in the discovery of pre-existing entities rather than their invention. While this view has intuitive appeal, particularly in theoretical physics, it faces what Benacerraf (1973) called the 'epistemological access problem': If mathematical objects exist outside space and time, how can human beings, as finite, embodied, spatiotemporal agents, come to know them?

Abandoning mind-independent mathematics does not, in itself, resolve the problem of access because the question is actually how meaning is accessed within formal systems. Hilbert's Formalism offers a well-accepted response by grounding mathematics as a self-contained system of symbols manipulated according to syntactic rules. Within this framework, mathematical truth is defined by formal derivability from axioms. Regarding the access problem, learning is often achieved through mastering symbolic manipulation within formal systems (e.g., a proposition is true if it follows logically from a set of axioms through valid inference). Learning mathematics, then, becomes the mastery of symbolic manipulations (procedural fluency) when operated correctly within a formal system, long before the discovery of meaning. In the context of quantum theory, formalism offers a rigorous mathematical framework in which a system's state is represented by a vector (the wave function) in Hilbert space, observables as operators, and measurement outcomes as probabilistic projections. Here, understanding of a quantum system is often reduced to procedural fluency with vectors, operators, and transformations, with limited representational access to the underlying relational structure.

While Hilbert formalism ensures precision and consistency, procedural fluency in symbolic manipulation does not guarantee access to the underlying structures those symbols encode. In quantum theory many central features resist straightforward reduction to independently specifiable parts. Entanglement, for example, shows that relational structure is not just a derived property but fundamental to the formalism: the behavior of a comnposite system cannot, in general, be reconstructed from its components alone. Within Hilbert space, one begins by assuming that systems have states represented as vectors and that their combinations follow tensorial rules. In quantum reality, however, the parts cannot be treated independently: the whole cannot be reconstructed just as the sum of its constituents (Aerts and Coecke, 1999).

The key issue here is not the choice of formalism, but how meaning is accessed within a representational system. In many contexts, knowing is equated with possessing a correct and consistent representation—symbolic or otherwise—and being able to manipulate it according to well-defined rules. However, such procedural competence does not necessarily ensure conceptual understanding. From an access-oriented perspective, knowledge involves more than the application of formal operations; it requires the ability to construct, interpret, and relate structural elements encoded in a representation. In this sense, the shift is toward accessible knowledge—the capacity to work with how elements compose and relate—a view that aligns with enactive approaches (e.g., Varela et al., 2016), in which understanding emerges through active engagement with structured environments.

This perspective finds a natural correspondence in diagrammatic approaches to quantum theory. In Quantum Picturalism, diagrams function as a formal, rule-governed representational language that makes the compositional structure of quantum processes explicit (Coecke and Duncan, 2011; Coecke and Kissinger, 2017). Instead of decomposing systems into independent parts, this pictorial approach treats processes as morphisms between systems, privileging *connectivity* and transformation over *objecthood*. This marks a shift from knowing through formal symbols, as in Hilbert's abstraction, to knowing through *composable processes*, adopting an enactivist perspective, where knowledge becomes a language of composable *relations—a mode of understanding rooted in creation rather than passive observation*.

In quantum theory, this approach is captured by process theories, formulated via category theory (Abramsky and Coecke, 2004; Coecke and Kissinger, 2017). A process theory is a mathematical and conceptual framework in which processes—transformations, interactions, and dynamics—are taken as primitive, while systems are defined by the roles they play as inputs and outputs of these processes. Central structural features of process theories include:

- Sequential composition: doing process *f* then *g* process yields a composite process *g*°*f*.
- Parallel composition / tensoring: processes may occur independently or in parallel.
- Identity / trivial processes: there is a no-op process that does nothing to a system.
- Symmetries or causal constraints: processes can have inputs and outputs; there may be constraints like “no-signaling”, or “causality”.

Category theory provides a precise mathematical language for such process-based framework. For example, in categorical terms, objects represent system types, while morphisms represent processes or transformations between them. Composition of morphisms captures sequential processes; identity morphisms represent trivial or null transformations. For physical theories, monoidal categories introduce a tensor product that formalizes parallel composition of systems and processes. Additional categorical structure—such as symmetry, dagger operations, and compact closure—supports duality, reversibility, and the representation of entanglement through diagrammatic “cups” and “caps”. These cups and caps represent Bell states and effects, which suffice to generate all entangled states and multi-partite operations in a †-compact category (Coecke, 2008). These structures allow one to reason rigorously about composite quantum systems and their transformations without presupposing a particular Hilbert-space realization. Importantly, any diagram for entangled quantum systems must be connected through at least one wire, while for unentangled quantum systems must be disconnectable. Furthermore, these diagrammatic representations naturally lift from pure-state to mixed-state quantum mechanics, where cups and caps between pure quantum channels and their adjoints represent the maximally mixed state and partial trace (Selinger, 2007; Coecke, 2008).

Diagrammatic calculi emerging from this categorical framework—most prominently in what has come to be known as QPic—replace symbols with string diagrams as the primary representational medium (Coecke and Kissinger, 2017). Crucially, this is not just a pedagogical or aesthetic choice. Diagrammatic representations make explicit the compositional and relational structure of quantum processes, allowing causal dependencies, information flow, and constraints such as no-signaling to be seen directly. Algebraic equalities correspond to topological equivalences of diagrams, and many foundational results—such as teleportation, unitarity, and no-broadcasting—admit proofs that are both more general and more explanatory than their operator-based counterparts.

This framework resonates strongly with process philosophy (Whitehead), which holds that processes are ontologically prior to independently defined objects. In categorical quantum mechanics, by contrast, objects function primarily as interfaces—placeholders marking where processes begin and end—while morphisms carry the substantive content. Knowledge, in this sense, is not the possession of a static symbolic description, but an understanding of how processes compose, constrain one another, and give rise to observable phenomena.

Seen in this light, diagrammatic approaches to quantum theory do not compete with Hilbert-space formalisms so much as they reorient what it means to 'understand' quantum phenomena. The shift is not just representational but also ontological because the focus shifts from states to processes, from objects to relations. The philosophical significance lies not in adopting a particular formalism, but in recognizing that certain mathematical languages are better suited to expressing a process—and, correspondingly, to supporting forms of understanding grounded in composition and relationalism.

Within this perspective, category theory can be understood as providing a formal ontology of processes. Objects function primarily as placeholders for the inputs and outputs of processes, while morphisms carry the substantive content, representing transformations, interactions, or evolutions. Table 2 identifies the advantages of this process-centered diagrammatic framework.

**Table 2:** Advantages of process-centered, diagrammatic framework.

Conceptual dimension	Process-centered diagrammatic framework
Explicit composition	Diagrammatic representations make composition explicit, rendering causal structure, dependencies, and constraints directly visible. Features such as no-signaling, locality, and complementarity often emerge transparently, rather than remaining implicit in algebraic or index-heavy formulations.
Diagrammatic proof styles	Categorical frameworks support proof styles in which reasoning proceeds diagrammatically or topologically. Such proofs are often more compact and conceptually illuminating than matrix-based calculations, emphasizing structural necessity over component-level manipulation.
Generality across theories	Many arguments hold across broad classes of process theories without assuming a specific Hilbert-space realization. This enables transferability across domains and conceptual unification spanning quantum physics, quantum information, and networked systems.
Higher-order processes	Categorical quantum mechanics naturally accommodates higher-order processes—processes whose inputs or outputs are themselves processes—as well as causal constraints and scenarios involving indefinite causal order. Category theory provides the formal tools required to rigorously articulate these structures.
Uniform Diagrammatic Theorems	Key results, including no-signaling, no-broadcasting, unitarity, and teleportation, admit diagrammatic proofs that apply uniformly across many models of quantum-like behavior, clarifying why such results hold rather than merely that they do.
Explicit information flow	Because compositionality is built directly into the syntax, diagrams display how processes depend on one another: which systems interact, which operations occur in parallel, and how information flows, avoiding the black-box character of operator-based approaches.
Alignment with physical reality	The process view aligns closely with interaction, temporal evolution, causation, measurement, and entanglement, avoiding the elevation of representational artifacts—such as basis choices or coordinate systems—to fundamental physical status.
Structural explanation	The diagrammatic paradigm promotes structural explanations: outcomes arise because networks of processes are composed in particular ways, providing deeper insight into why physical effects occur beyond procedural or computational accounts.

**Figure 2:**
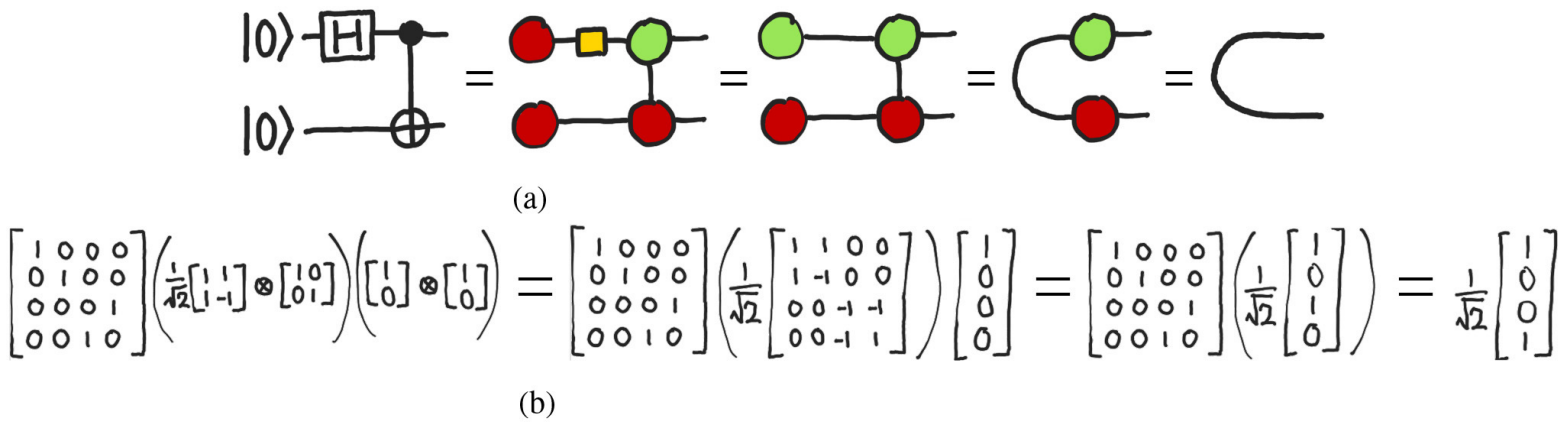
Comparison of Bell state calculations: top using diagrammatic QPic notation, bottom using Hilbert space formalism (matrix notation). **(a)** Bell state circuit calculated using QPic. **(b)** Matrix calculation of the same circuit.

Here, we next juxtapose a single quantum process expressed in different representational regimes to make the underlying frameworks visually explicit. Figure 2 presents two calculations verifying the quantum circuit for the standard construction of the Bell state. This is the simplest and first example of an entangled quantum state that students typically encounter.

Although both these calculations, diagrammatic and matrix-based, can be considered teachable to young learners, the matrix-based approach is not transferable to large quantum computations. This limitation is inherent to scalability: a quantum computation on *n* qubits is described by exponentially large matrices consisting of 2^*n*^ by 2^*n*^ complex numbers. Many quantum concepts require at least 3 qubits to approach, necessitating size 8 by 8 matrix calculations which are cumbersome and easy to make mistakes on.

**Figure 3:**
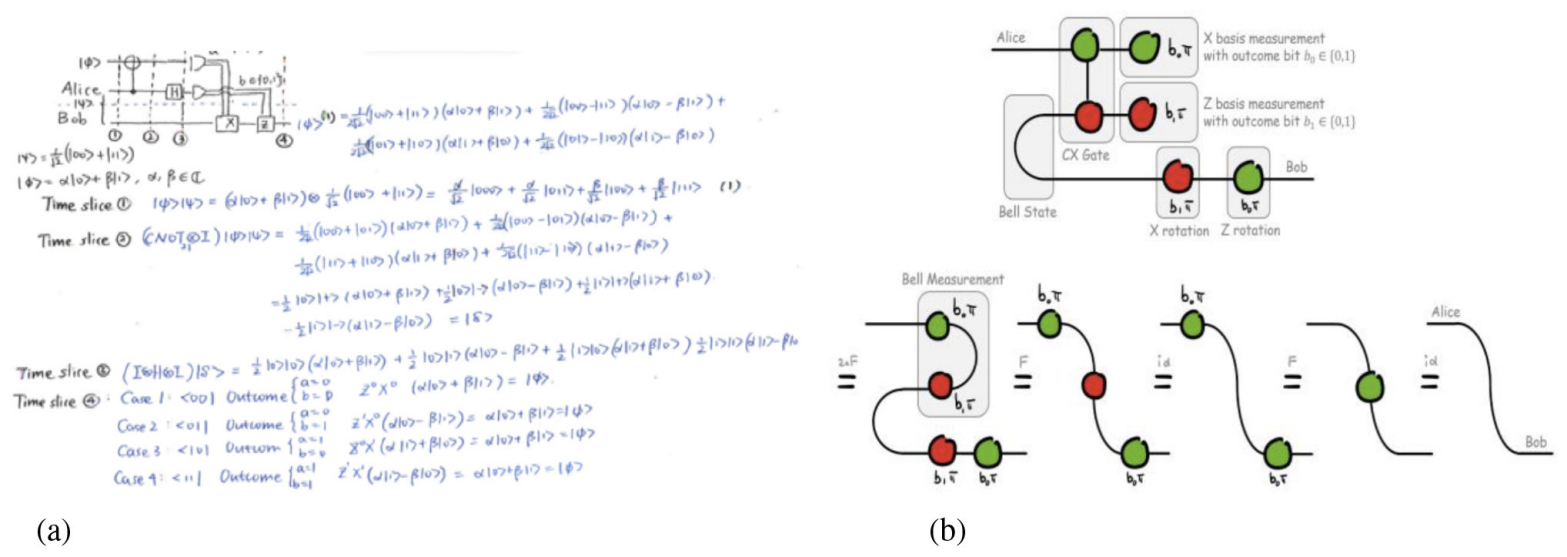
Comparison of quantum teleportation protocol calculations reprinted from Dündar-Coecke et al. (2023). Left: Using Hilbert space formalism (bra-ket notation). Right: Using diagrammatic QPic notation. **(a)** Bra-ket calculation of quantum teleportation protocol. **(b)** Diagrammatic calculation using QPic.

Similarly, for another advanced topic—quantum teleportation, Figure 3 illustrates the same quantum protocol through two different lenses, emphasizing that one representation doesn't supersede the other, but rather to expose how different formalisms foreground different aspects of the same physical reality. On the left, the traditional Hilbert-space approach relies on vectors, operators, and matrix gymnastics—yet often conceals the relational and compositional structure of quantum processes behind layers of symbolic manipulation. On the right, the QPic diagrammatic formalism translates the same process into intuitive visuals without compromising mathematical correctness: wires, boxes, and topological connections make entanglement, measurement, and communication immediately visible.

These side-by-side comparisons are intended to contrast how the calculation is organized and what structural features are made explicit. The comparison thus illustrates how ontological commitments about what is taken to be primitive—states or processes—translate into different modes of calculation and explanation.

What is at stake, therefore, is not just a difference in notation, but a difference in epistemic access. In the symbolic algebraic framework, understanding is frequently mediated through symbolic manipulation and *post-hoc* interpretation, whereas in the diagrammatic framework, many constraints and dependencies are apprehended directly through spatial and topological relations. Entanglement, for instance, appears not as a property of a state vector but as a structural feature of the network of processes; measurement is represented not as an abstract projection operator but as an interaction that reshapes the flow of information.

We now focus our attention from advantages to consequences, examining how different ontological commitments shape representational practice. Table 3 places Hilbert-space and diagrammatic formalisms side by side, revealing how a representation determines what is made explicit, what is left implicit, and how reasoning proceeds.

**Table 3:** Representational consequences across comparative aspects in Hilbert-space vs. diagrammatic formalisms.

	Hilbert-space formalism	Diagrammatic (QPic) formalism
Nature of processes	Quantum processes represented via linear algebra: state vectors, tensor products, linear operators, and matrices.	Quantum processes represented diagrammatically as morphisms; boxes denote processes and wires denote systems.
Compositionality	Sequential and parallel composition encoded algebraically through tensor products and index structure.	Sequential and parallel composition explicit in diagram topology; compositional structure is directly visible.
Information structure	Causal dependencies and information flow are implicit and must be reconstructed symbolically.	Causal structure, entanglement, measurement, and information flow are directly visible in the diagrammatic structure.
Reasoning	Reasoning proceeds via symbolic manipulation, matrix algebra, and component-wise calculation.	Reasoning proceeds diagrammatically or topologically, emphasizing structural necessity over calculation.
Generality of arguments	Arguments are typically tied to specific Hilbert-space realizations and representations.	Arguments apply uniformly across broad classes of process theories, independent of a fixed Hilbert-space realization.
Cognitive load	High cognitive load due to index tracking, symbolic bookkeeping, and algebraic inference.	Lower cognitive load; promotes visual, relational, and structural modes of understanding.
Explanatory emphasis	Prioritizes numerical prediction; structural relations are obscured.	Promotes structural explanation, clarifying why outcomes arise from compositional architecture.

Although both paradigms are formally equivalent in their expressive power, they differ markedly in how that power is cognitively assessed. These differences do not concern what can be expressed, but rather how understanding is formed, motivating the discussion in the next section.

## 3 Cognitive advantages: an intuition-perception-comprehension hybrid

Classical philosophical traditions positioned mathematics as a purely formal enterprise, from Hilbert's formalism to Carnap's logical positivism (Carnap, 1959), treating it as a symbolic system governed by explicit rules—a self-contained domain of signs. Within this framework, mathematical entities such as numbers, sets, and operations are manipulated through a precise logical syntax, where truth emerges not from observation but from internal consistency—the archetype of formal reasoning—abstract and rule-bound (Whitehead and Russell, 1910–1913; Shapiro, 2000; Sieg, 2013).

In recent years, there has been growing recognition that, although symbolic mathematics has long been seen as the language of physics, it is not necessarily the language through which the mind thinks about the physical world. While symbolic formalisms are highly effective at capturing physical regularities and enabling precise prediction, their descriptive success does not imply that they mirror the cognitive mechanisms by which such knowledge is formed, understood, or manipulated. For example, symbolic operations alone have proven insufficient to mediate a genuine dialogue between perceptual experience and abstract cognitive processes. This limitation becomes especially salient in mathematical thought—and in quantum theory in particular—where understanding appears to depend on a dynamic feedback loop between perceptual intuition and formal abstraction, preserving the dual character of mathematical knowledge as both discovered in nature and invented by the mind. It must be *discovered* because mathematical relationships may exist independently of human minds, revealing themselves through systematic exploration of the physical world; and it is also *invented* because the very languages, formalisms, and representational systems through which we access these relationships are human constructions—products of our cognitive architecture and linguistic practices.

Evidently, the human mind draws on a diverse repertoire of cognitive tools in both discovery and invention (Tversky, 2013, 2019; Kahneman, 2011). Understanding mathematical thought therefore requires attending not only to the formal structures that describe physical reality, but also to the cognitive mechanisms through which those structures become intelligible, learnable and usable. Evidence from cognitive science reveals a far more dynamic picture of the contexts in which the mind is best at, challenging the longstanding detachment of mathematics from perceptual-cognitive processes. In this section, we discuss some of the most relevant cognitive approaches that shed light on this relationship.

### 3.1 Bridging human intuition and quantum abstraction through this hybrid

Traditional approaches to teaching quantum theory, grounded in algebraic operations within Hilbert spaces, pose exceptional challenges to human mind. Such methods rely on dense formal systems that demand extensive working memory, sequential reasoning, and the ability to navigate complicated abstractions. Even in learning algebra alone, learners often need to translate external notation into an “inner code” that the mind can manipulate (De Cruz and De Smedt, 2013). In quantum mechanics the cognitive demand is even greater because learners must retain, transform, and recombine multiple symbolic derivations within working memory, often struggling to extract coherent structural insight from sequences of procedural operations.

This limitation raises a deeper question: how can quantum mathematics be structured so that it aligns with human cognitive strengths? Addressing this question requires examining the nature of symbolic thought and acknowledge the long history of how humans have learned to internalize, reproduce, and manipulate mathematical relations—from basic intuitions of numerosity to higher-order abstract operations through training.

Crucially, this process has never been completely abstract. To master mathematical reasoning, the mind must navigate at least two complementary dimensions of symbolic thought: symbols as a reference to objects and symbols as representations of procedures (Sfard, 1991). A symbol such as π, for example, can refer to a numerical entity 3.14159265⋯  yet it also represents an operation: in Euclidean geometry, π expresses the process of dividing a circle's circumference to its diameter. This dual function is the central to the power of mathematical notation. By stabilizing fleeting mental operations, symbols convert transient cognitive acts into manipulable objects of thought. Mathematics, in this sense, is not just a language of abstraction but a cognitive technology: one that extends the mind's capacity to perceive, construct, and transform structure.

Over centuries, this technology has been refined through symbolic compression. Operations once expressed verbally were condensed into signs like +, √, or ÷, enabling abstract processes as tangible cognitive objects that scaffold the development of higher order reasoning, including algebraic and analytical thought De Cruz and De Smedt (2007). Yet symbolic compression comes at a cost: as symbols become increasingly detached from perceptual grounding, they risk obscuring the very structures they were designed to reveal.

More recent cognitive accounts make this limitation explicit. Intuitionism, for instance, offer a bold rethinking of how mathematical knowledge is constructed and understood. Proponents of this view position mathematics as an active, living process of construction, suggesting that mathematical knowledge is meaningful only when it can be mentally constructed by the thinker—an idea that directly challenged the Platonist view of mathematics as a realm of objective, mind-independent truths (Brouwer, 1913). Drawing on embodied cognition and enactivism, mathematical concepts do not float above/beyond perception and action, but instead rooted in the body's interaction with the world—through perception, sensory-motor activity, and relational cognition (Lakoff and Núñez, 2000; Núñez, 2017). Numbers, operations, and geometrical forms are thus understood as evolving mental models shaped by interaction with structure, relation, and pattern.

Empirical research in mathematical cognition consistently shows that understanding does not emerge from symbolic manipulation alone, but from the integration of perceptual and spatial processes with the interplay between the concrete and the abstract (Butterworth, 1999; Lakoff and Núñez, 2000; Dehaene, 2011; De Smedt et al., 2013; Núñez, 2017). Human thought is not confined to linear symbolic manipulation, it is inherently relational, visual, and dynamic—echoing both Euclidean geometric intuition and Leibniz's vision of a *characteristica universalis*, a calculus grounded in relational thought.

The issue with pure symbolic reasoning in the quantum realm is that it linearizes thought, forcing inherently dynamic non-linear relations to unfold sequentially step by step and in doing so, it fragments structural coherence. The human mind, by contrast, does not operate as a serial processor of symbols; it excels at perceiving wholes, mapping relations, and integrating patterns in parallel. Visual and spatial systems are tuned for *structural reasoning*, for seeing how parts cohere within a system, rather than parsing strings of symbols (Monti et al., 2012; Slotnick et al., 2005; Tolmie and Dündar-Coecke, 2020; Mock et al., 2018).

It is against this cognitive backdrop that the shift introduced by QPic and more generally, by graphical algebraic approaches, becomes significant. In classical mathematics, the fundamental unit of reasoning is the *symbol* corresponding to the *object*; in QPic, it is the *diagram*—a compositional morphism/processes. Meaning no longer emerges solely from syntactic manipulation, but from structural connectivity. Such a shift transforms not only how we calculate, but also how we conceptualize knowledge, mirroring a relational, process-oriented view in which physical systems are understood as networks of interaction rather than isolated entities. By foregrounding relations, QPic reduces the Cartesian divide between observer and observed, and between computation and comprehension.

Because the form of mathematics we use shapes the kind of truths we can access, a world governed by deep relationality demands a corresponding cognitive framework. Gestalt principles provide a cognitive rationale for this alignment, explaining how the mind organizes perceptually grounded structures and revealing the advantage embedded in QPic's compositional logic. The roots of this compositionality trace back to classic Gestalt principles—grouping, continuity, similarity, proximity, Prägnanz (simplicity), and common fate (Wertheimer, 1923; Koffka, 1935): Rather than perceiving isolated fragments, the mind apprehends relations among elements. QPic leverages this tendency by presenting quantum processes as holistic perceptual structures, enabling learners to intuit relational coherence before engaging with formal syntax. When elements move or transform together, the mind perceives them as unified (*common fate*); when information is incomplete, it fills the gaps intuitively, restoring coherence from continuity and structural cues.

While learning QPic, learners benefit from engaging with visually and metaphorically grounded components—such as spiders, cups, and wires, etc.—rather than relying solely on symbolic interpretation. This resonates with conceptual metaphor theory, which shows that abstract concepts are often grounded in mappings from sensorimotor domains, scaffolding understanding by linking unfamiliar structures to perceptually accessible forms. This perspective is also consistent with hierarchical models of visual cognition [see Treisman and Gelade (1980)], particularly the idea of feature integration, in which local perceptual elements, such as directionality or causal flow, progressively combined into coherent wholes.

Taken together, these perspectives point to a broader evolutionary insight: relational, visual, and causal reasoning constitutes one of humanity's earliest cognitive technology—a survival mechanism that predates language itself (Donald, 1991). QPic draws on this ancestral capacity, harnessing visuospatial processing to represent transformation and relational structure in ways that are perceptually natural. In doing so, QPic reconnects quantum mathematics with the brain's intrinsic architecture for understanding that symbolic notation alone cannot achieve.

### 3.2 Cognitive pathways are potentially distinct in learning diagrammatic algebra: insights from behavioral studies

Across multiple experiments and teaching practices, we have observed that calculations in diagrammatic algebra engage multiple cognitive pathways simultaneously. One possible explanation is that the visual-compositional nature of the formalism reduces cognitive load: once learners internalize its compositional rules, they can mentally simulate quantum processes through pattern-recognition, perceptual grouping, and intuitive structural mapping—mental operations that allow relational structure to be grasped holistically rather than assembled sequentially as in symbolic computation.

One way to characterize these cognitive pathways is to examine how learning unfolds across symbolic and diagrammatic modes of reasoning. Bloom's Taxonomy, a widely used framework in educational psychology, provides a useful lens for this analysis by describing learning as a progression from basic encoding and recall to higher-order mental processes (Bloom et al., 1956; Anderson and Krathwohl, 2001). In its revised formulation, the taxonomy distinguishes six levels of cognitive engagement—*Remember, Understand, Apply, Analyze, Evaluate*, and *Create*—providing a descriptive lens for examining how instructional practices distribute cognitive effort across different forms of understanding. Here, it can be used as an analytical tool to assess whether pedagogical approaches primarily support lower-level procedural learning or facilitate access to higher-order conceptual reasoning.

When examined through this taxonomy, a substantial body of empirical research in physics and quantum education indicates that traditional, symbol-dominated instructional methods concentrate learner activity in the lower tiers of the hierarchy.

**Figure 4:**
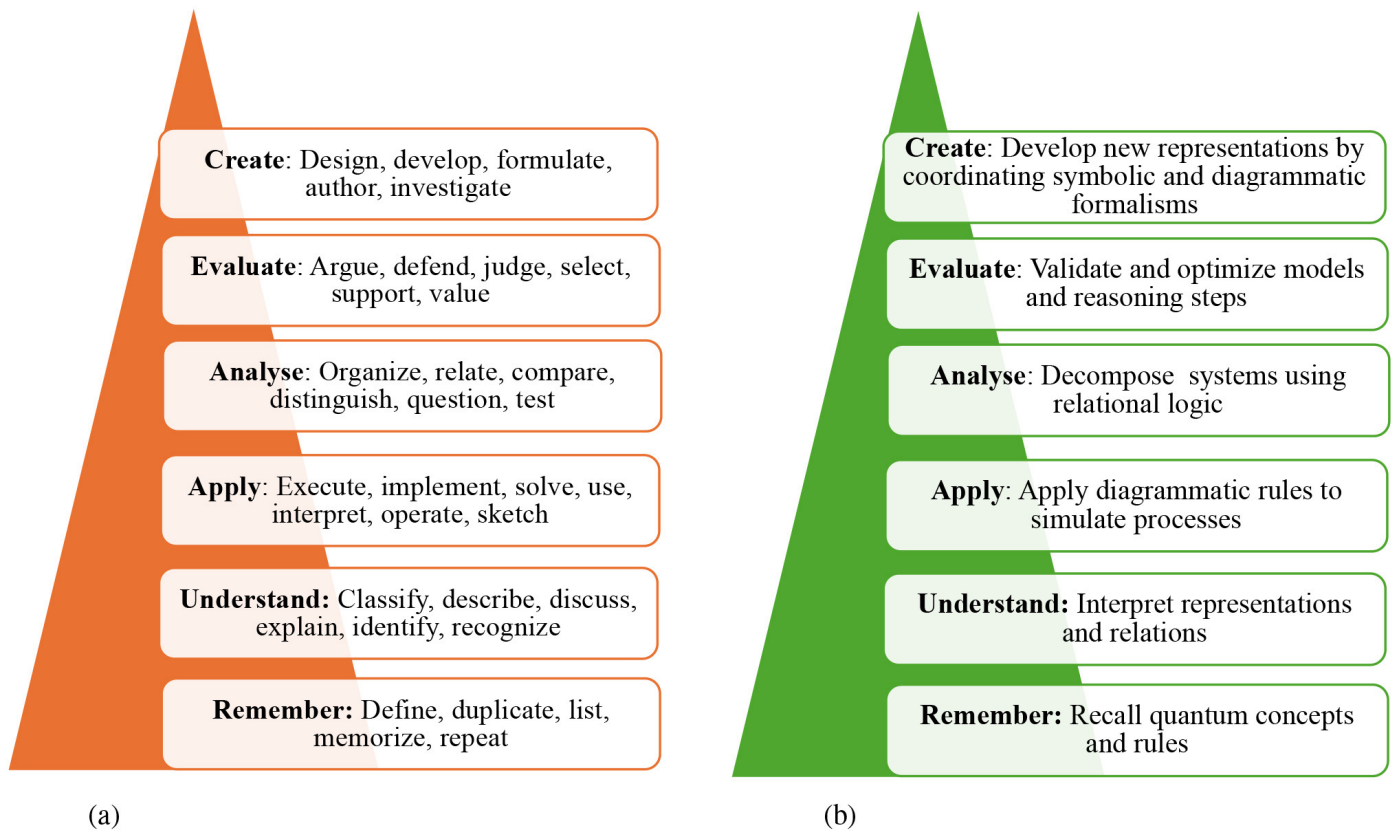
Comparison of the hierarchical structure of Bloom's Taxonomy (left) with its adaptation for QPic-based learning (right). The QPic framework reconfigures the hierarchy into a recursive and integrative process. Rather than isolating cognitive operations into discrete stages, QPic enables learners to engage multiple cognitive operations simultaneously and iteratively, without diluting mathematical rigor. **(a)** Original Bloom's hierarchy of learning. **(b)** Adapted Bloom's Taxonomy for QPic.

Empirical studies report that learners spend most of their time mastering symbolic manipulation, procedural problem sets, and rote conceptual recall (Krijtenburg-Lewerissa et al., 2017; Scottish Qualifications Authority, 2024)—activities that sit primarily within the *Remember* and *Understand* levels illustrated in Figure 4a. The consequence is a cognitive bottleneck: even advanced learners may develop procedural fluency without gaining structural insight, as their reasoning remains sequential, algebraic, rule-based rather than relational.

Singh (2008) reviews research on students' reasoning, problem-solving, and metacognition in upper-level quantum mechanics, emphasizing that students commonly rely on algorithmic manipulation and struggle to coordinate representations and interpret formalisms—again consistent with the bottleneck before higher-order reasoning becomes robust. Similarly Johnston et al. (1998) presents empirical data documenting student difficulties (misconceptions, representational confusion, weak coherence between maths and meaning), which helps justify the “bottleneck” claim in Bloom terms. Further, studies reviewing research-based approaches to quantum teaching and learning (Donhauser et al., 2024) compare learning efficiency across different instructional designs and highlight a strong trend toward introductory treatments of the broad and differentiated field of quantum science.

German et al. (2023) presented another diagrammatic reasoning framework, the Quantum Abacus framework, and compared it to the QPic framework through proofs of the GHZ state and quantum teleportation. QPic's graph-rewriting techniques revealed process invariance and systemic relations directly, while the Abacus approach offered a “slow-motion replay” of sequential evolution. By compressing temporal and procedural sequences into structural form, diagrammatic reasoning promotes conceptual synthesis, relational understanding, and creative manipulation, rather than rote repetition.

Effective learning typically involves a recursive process that engages multiple cognitive levels—from basic recall to analytical reasoning, and higher order creative synthesis. In this context, teaching quantum theory exclusively through symbolic methods presents a substantial challenge. This difficulty is amplified by the mathematical prerequisites of symbolic formalisms, such as linear algebra and complex numbers, which can delay access to conceptual understanding, particularly at early stages of instruction (Krijtenburg-Lewerissa et al., 2017).

In contrast, experience with teaching QPic shows that the diagrammatic paradigm disrupts traditional symbolic-bound reasoning, it shifts the locus of understanding from procedural execution to active meaning-making. Comprehension emerges organically through the dynamic interplay of perception and structure: perceptual recognition helps reveal patterns, recognition enables systematic transformation, and transformation gives rise to creative construction, as learners generate their own diagrams as part of problem-solving steps. In doing so, lower-level perceptual and procedural skills are progressively integrated into higher-order conceptual insights, allowing learners to *analyze, evaluate*, and *create* relational systems (Figure 4b) much earlier in their training than would be possible with purely algebraic methods. Of course, this does not imply that such higher level capabilities are exclusive to diagrammatic thinking, rather, experienced learners often develop them by flexibly coordinating symbolic and non-symbolic formalisms.

**Figure 5:**
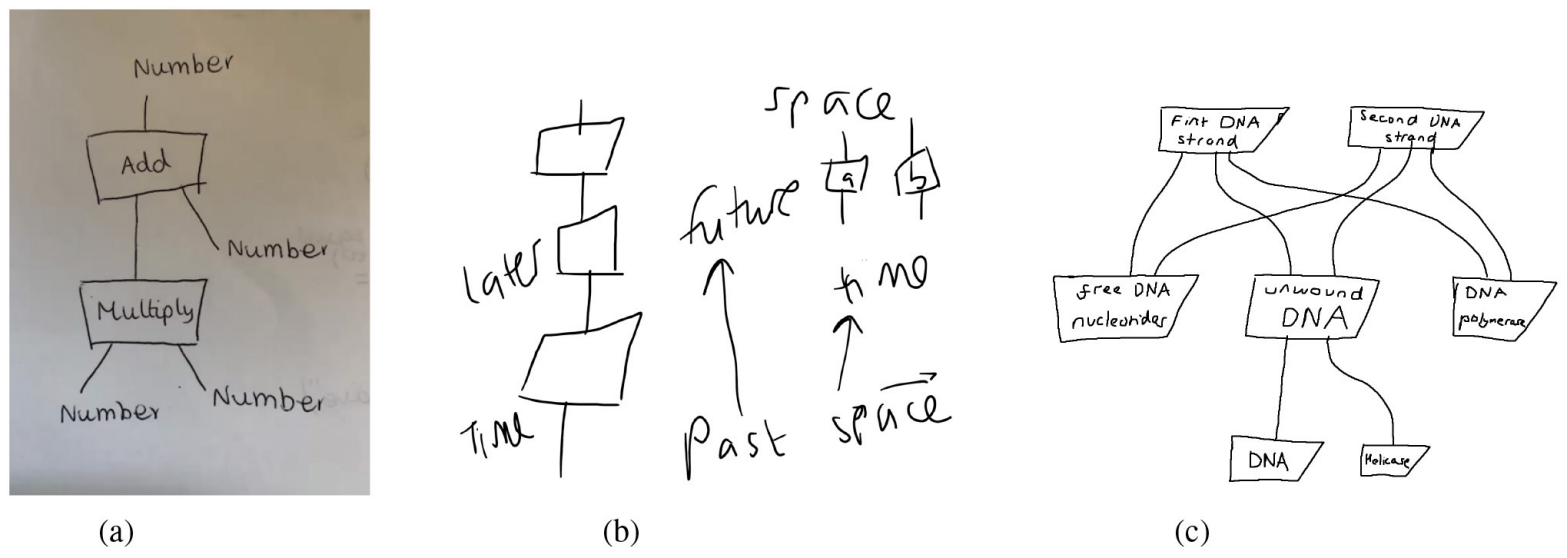
At the end of the first week's tutorial of the QPic course, every student drew and shared a diagram they drew in under 5 min (Dündar-Coecke et al., 2025). These three students' diagrams exemplify conceptualizing that mathematical operations can be represented as processes (Left), that processes can be occur in sequence at the same space or in parallel at the same time (Center), and that different processes are composable together to form a more complex process (Right). **(a)** Arithmetic. **(b)** Space and time. **(c)** DNA replication.

We consistently observed that after only 1–2 h of course instruction, the students develop familiarity with mental organization of concepts in a two-dimensional as opposed to one-dimensional representation, drawing diagrams such as in Figure 5. Crucial in quantum information theory, the interchangeable properties of space and time are natural to express through diagrammatic reasoning, facilitating ease of transition from informal exposure to quantum concepts to re-understanding the same diagrams interpreted with full mathematical formality. Indeed, the quantum teleportation protocol is taught twice in the QPic curriculum in order to reinforce the concepts, adding more specificity and complexity of explanation on each revisit. At the end of the course, the students exhibited indications of ability to *create*, the highest level of Bloom's Taxonomy in Figure 4: Students performed highly on the final exam, including the most conceptually difficult question, which tasked them to create multiparty quantum communication protocols and prove their correctness (Dündar-Coecke et al., 2025).

This integrative potential aligns with well-established accounts of cognitive mechanisms, such as *mental animation* highlights the mind's capacity to simulate dynamic processes (Hegarty, 2004); *spatial scaffolding* demonstrates how perceptually structured information can reduce cognitive load and clarify complex patterns (Tversky, 2013); *analogical transfer* provides a mechanism for mapping structural relations across contexts (Gentner, 1983). Future research should explore how the perceptual and spatiotemporal components of diagrammatic formalisms enhance engagement with abstract quantum concepts, offering an empirically grounded account of the cognitive advantages suggested by these theoretical perspectives.

These observations are consistent with broader research on cognitive architectures, which seeks to model core cognitive abilities—including perception, attention, memory, and reasoning—within integrated computational frameworks [see Kotseruba and Tsotsos (2018) for a review]. A central insight from this literature is that different representational paradigms—symbolic, sub-symbolic, and hybrid—offer distinct trade-offs between formal precision and interpretability: while symbolic systems support structured reasoning, they often struggle with handling perceptual and context-sensitive processing, whereas distributed approaches enhance flexibility but at the cost of reduced transparency. From an access-oriented perspective, these trade-offs highlight the importance of representational formats that support both formal reasoning and direct engagement with structure—an alignment that Qpic is well-positioned to provide.

## 4 Educational merits: innovating with tech-driven pedagogy

Prior to the development of QPic, a fully diagrammatic mathematical formalism had not been explored or implemented in education. While diagrams have long served as auxiliary representational tools, they have rarely functioned as the primary medium through which mathematical structure, inference, and problem solving are taught and learned. In this section, we examine how learning practices can be fundamentally re-engineered through cross-disciplinary integration, and assess the broader socioeconomic implications of such tech-driven pedagogy.

### 4.1 Cognitive elitism vs. inclusive workforce development: empirical evidence for early skills development in QIST

Building on the promising outcomes of the UK pilot, 10 additional educational QPic experiments have been completed and are currently underway across multiple countries (including UK, Pakistan, Greece, Türkiye, Ghana, Sweeden, Australia, and the USA). Each study is conducted under independent academic supervision to ensure methodological rigor and sociocultural and contextual validity. To varying degrees, these parallel studies mirror the original experimental protocol and use the course materials developed during the UK pilot, enabling cross-comparison and the construction of a robust meta-dataset.^1^ Collectively, these studies aim to assess the extent to which diagrammatic reasoning, when used as a pedagogical framework, facilitates early acquisition of complex QIST concepts. The integrated metadata, expected to be completed by 2027, will provide critical insights into how such methodologies can foster accessible, scalable, and inclusive early skill development in one of the most cognitively demanding scientific domains.

Each experiment had its unique setting, yet they were supported by the QPic team to obtain an objective view of whether the QPic research program constitutes a genuinely tech-driven pedagogy, and crucially, whether its overarching goal—to foster early skills development in complex STEM domains—can be empirically substantiated. More critically, it sought to determine whether such an approach can help reduce *cognitive elitism* and promote a more inclusive, creative, and quantum-literate workforce.

One might argue that such a vision is ambitious and necessarily long-term. This is indeed the case. And also, there exists a central obstacle rooted in the historical structure of today's school systems: modern schooling is largely inherited from the industrial era, having been designed to optimize standardization, efficiency, and the mass-production of a uniformly skilled workforce. Learning is typically organized around linear curricula, age-based progression, and assessment regimes that privilege symbolic manipulation and procedural compliance.

Since the post-Second World War period, successive waves of educational reform have further reinforced this orientation by progressively dismantling the earlier tripartite structure of education, in which different forms of intelligence are cultivated across three pathways: (1) theoretical, (2) practical, and (3) a combination of both theoretical and practical.

**Figure 6:**
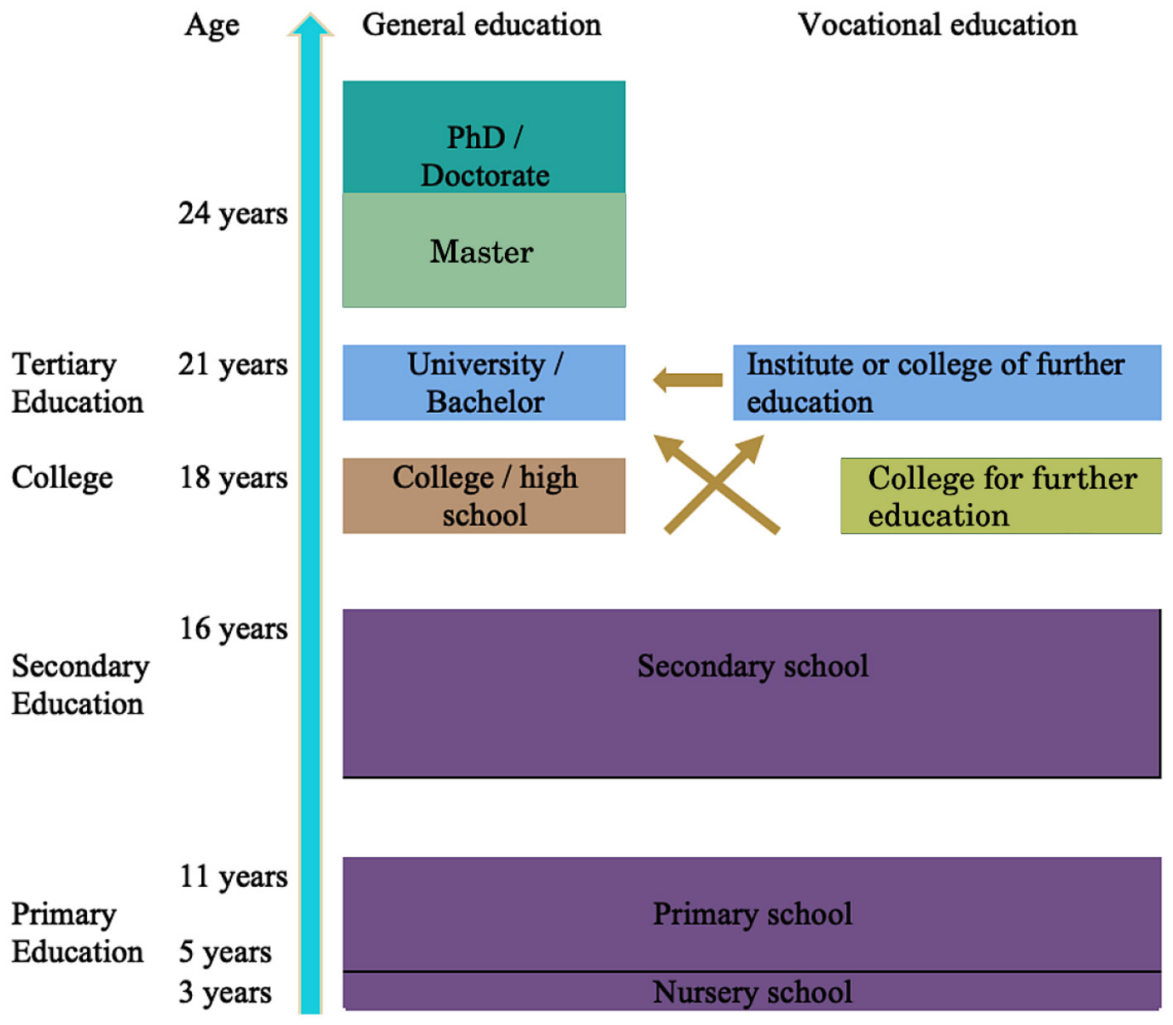
A standard (tripartite) model of educational stratification for talent differentiation.

These reforms were shaped by modern psychological research that initially reinforced fixed notions of intelligence (as exemplified by the Trinity Psychology of Talents Aytaç, 1980). Despite alternative research proposing instead that abilities are dynamic and particular skills can be developed given the right opportunities in critical periods of life (e.g., Piaget, Vygotsky), the legacy of the tripartite system has persisted and is still the backbone of the tracking and streaming practices found in many education systems, as shown in Figure 6. Contemporary schooling continues to channel students along differentiated trajectories that mirror class-based hierarchies—those identified as “academically gifted” climb up toward higher education and leadership roles, while others are directed toward more practical or vocational routes.

The tripartite structure not only implemented social Darwinism, but in practice it also operationalised *cognitive elitism*, embedding the notion that talent and social value are inherently stratified at the core of educational practices. Despite successive waves of school reforms, the architecture of this hierarchy remains largely intact. Contemporary schooling continues to mirror the early 20th-century model, channeling students into differentiated tracks: theoretical and abstract domains for those perceived as intellectually elite, vocational paths for those labeled as practically skilled, and intermediary routes for those navigating between both (Aytaç, 1980; Bowles, 1971). Such structures sustain what Richardson and Bourdieu (1986) termed the *reproduction of cultural capital*, ensuring that opportunity is often inherited rather than cultivated.

On the other hand, the demands of quantum-AI driven technological transformation call for highly differentiated forms of talent and do not align with classical pedagogies that foreground static knowledge, linear reasoning, and uniform outcomes. Initiatives like QPic pose a strategic question: Can we reconfigure access to high-level conceptual work, enabling a broader range of learners to engage with cognitively demanding fields earlier in their educational journey? Such an approach challenges the systemic assumption that only a small intellectual elite can engage with the complexities of quantum theory. Inclusive frameworks of this kind are no longer just educational ideals; they are strategic imperatives for cultivating a future-ready workforce. At present, entry into the mathematical foundations of QIST typically occurs in late undergraduate years, with genuine mastery achieved only at the postgraduate or doctoral level. By that stage—often between 25s–30s—graduates are entering a workforce already transformed by the accelerating evolution of quantum and AI technologies. This temporal lag between *learning* and *innovation* raises critical questions about educational return rates and social equity.

### 4.2 Tech-driven pedagogy as a catalyst for STEM ecosystem growth and future readiness

The *return rate of education* has long been a key indicator from the economics of education perspective, traditionally measuring the *private and social returns* of additional years of schooling—most often quantified through higher lifetime earnings, productivity, and employability (Psacharopoulos and Patrinos, 2018). Within this framework, education functions as an investment in *human capital* (Becker, 1918), where learning yields measurable economic dividends over time. However, in a world now defined by exponential technological change, this classical cost–benefit model is also under increasing strain. The logic of incremental educational investment—“more years = higher earnings”—no longer maps cleanly onto the realities of a rapidly transforming technology-driven job market. The time lag between learning and its application has narrowed because the half-life of skills has changed dramatically (World Economic Forum, 2023). As a result, relevance, adaptability, and cognitive transfer have emerged as the new currencies of educational value (Brynjolfsson and McAfee, 2014; Autor, 2022).

In this transformed landscape, the question is no longer simply *how long* individuals are educated, but *how effectively* they are equipped to participate in and shape dynamic, tech-centered ecosystems. The integration of quantum, AI, and emerging technologies is driving an unavoidable paradigm shift in pedagogical realm—what and how we must teach. This shift demands an epistemic redesign—a tech-driven pedagogy—potentially *interactive, data-informed*, and *cognitively adaptive*. Such a model moves beyond content transmission toward *conceptual modeling, creative reasoning*, and *cross-domain synthesis*—competencies essential for navigating complex, evolving problem spaces (Fischer et al., 2020).

The field *andragogy* resonates strongly with this new reality, positioning learners as active leaders in directing their own learning trajectories Knowles and Associates (1984); Dündar-Coecke (2020). The rapid technological changes have accelerated this shift from pedagogy to andragogy, gradually eroding formal institutions' long-held monopoly over access to technical knowledge. In its place, hybrid learning ecosystems, largely driven by tech companies in quantum AI and emerging technologies and platforms such as hackathons, maker spaces, online laboratories, are redefining what counts as expertise. Learning-by-doing, microcredentialing, and problem-specific learning now rival traditional degree pathways in both speed and labor-market impact (OECD, 2022; McKinsey Global Institute, 2023).

**Figure 7:**
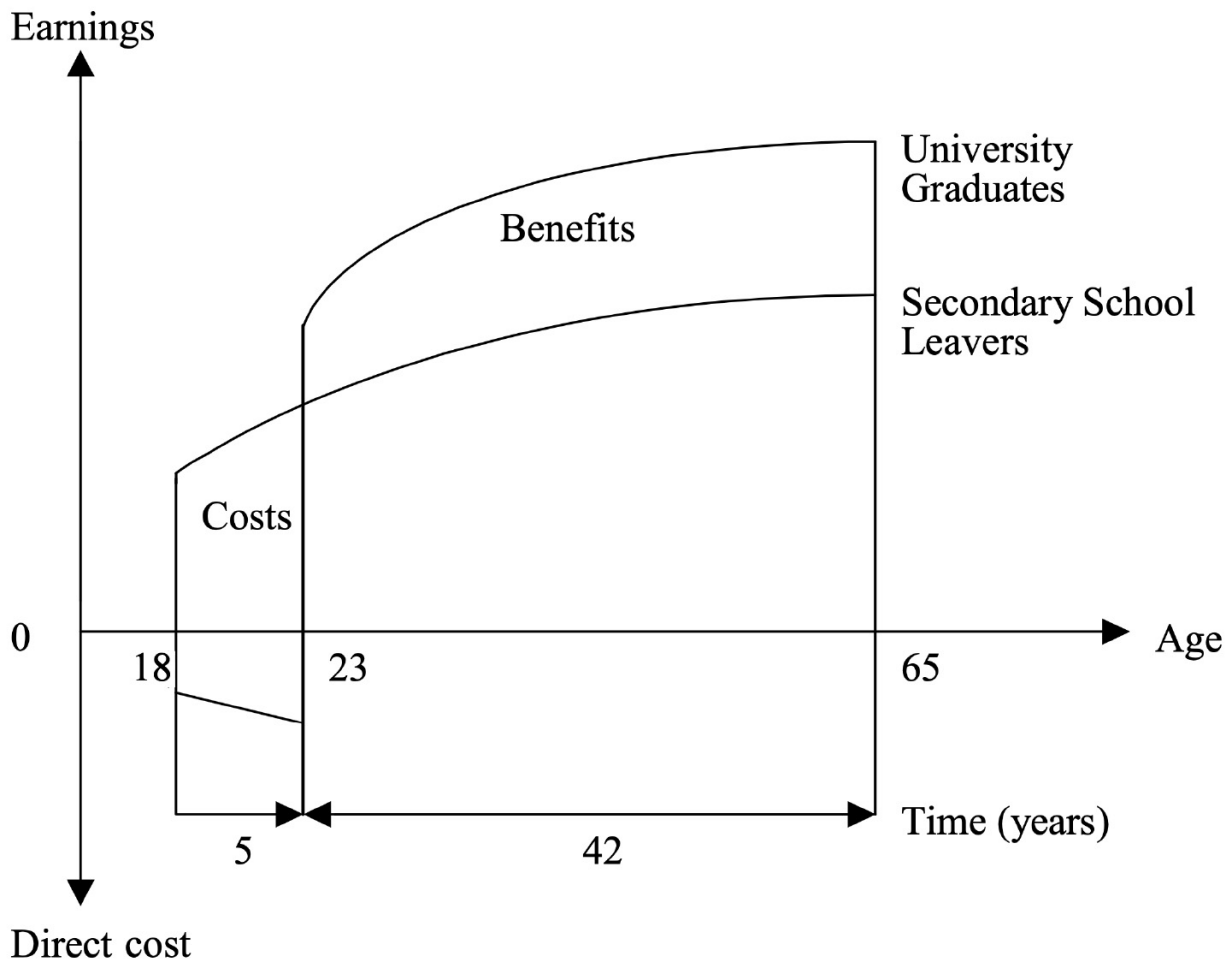
Stylized age-earning profiles (adapted from Psacharopoulos, 1995: the Profitability of investment in education).

Consequently, the conceptual and measurement frameworks used to analyze human capital investments—such as the classical *age–earnings profiles* (see Figure 7) comparing university graduates to secondary school leavers—are undergoing disruption. It is increasingly unclear whether individuals' income and social mobility will continue to correlate primarily with their position in the educational hierarchy.

The emerging tech driven economy rewards specific, scarce, and application-driven expertise—particularly in domains such as AI, quantum computing, and advanced data analytics. Professionals in their twenties and thirties who master these niche skills are increasingly outpacing senior academics and industry veterans trained under older paradigms, signaling a profound reconfiguration of how education, expertise, and economic value intersect.

This shift also reframes the role of education itself. As Bowles and Gintis (1976) first observed, much of the effect of schooling on earnings may derive not from the direct enhancement of cognitive skills but from the cultivation of *non-cognitive* attributes through educational participation. For example, in large-scale predictive models of hourly earnings, composite measures of non-cognitive traits (e.g., adaptability, initiative, resilience) yield regression coefficients four times larger than those for cognitive test scores, and 50% larger than those for years of schooling (Heckman and Kautz, 2012). This highlights that in a tech-driven market, traits such as curiosity, flexibility, creative problem-solving, and access to tech knowledge are becoming more valuable than domain-specific knowledge offered by traditional institutions.

These trends are also reflected in labor market outcomes. OECD data show that in the UK, for example, degree holders in engineering, manufacturing, and computing earn the highest median wages, while graduates in arts, humanities, and social sciences rank among the lowest (OECD, 2024). This widening income differentiation reflects the new “technological return rate” of education—one governed less by institutional prestige and more by the alignment between educational content, where economic value is increasingly determined by cognitive adaptability, and emergent technological ecosystems (Source: https://data.oecd.org/eduresource/education-spending.htm).

## 5 Discussion

In this work, we examined the merits of diagrammatic formalism from a holistic and integrative perspective, structuring the analysis around three interrelated dimensions: epistemological, cognitive, and educational. Together, these dimensions articulate not only how diagrammatic formalisms function, but why they matter for understanding, learning, and knowledge production in contemporary science.

- Section 2 focused on the *epistemological value*—what we know and how—situating the formalism within broader debates in the philosophy of mathematics and physics, and contrasting object-centered and symbol-dominated approaches with process-centered and diagrammatic modes. The aim was to clarify what it means to know in a framework where relations, transformations, and composition are primary.
- Section 3, outlined the *cognitive advantages* of intuitive-perceptual-comprehension hybridization. Here, we drew on cognitive science and learning theory to explain how diagrammatic and relational representations align with human perceptual and reasoning capacities. This section focused on how understanding emerges through visualization, interaction, and constructive manipulation, enabling learners to grasp complex quantum structures earlier and with reduced cognitive overhead.
- In Section 4, we discussed *educational merits* to innovating with the same pedagogical tools driving technology. This section explored how such representational frameworks can inform the design of future learning environments that integrate AI, simulation, and interactive tools. Rather than treating pedagogy as a downstream application, we argued that formalism itself can function as a pedagogical technology—reshaping curricula, assessment, and learner trajectories in ways better suited to contemporary scientific and technological demands.

The central question raised by this work is not whether quantum theory can be taught differently, but whether our prevailing conceptions of understanding are adequate for the kinds of knowledge contemporary science now demands. Quantum phenomena expose the limitations of object-centered, symbol-dominated epistemologies, revealing instead the need for modes of reasoning that are relational and processual. What is challenging here is not a curriculum, but an epistemic inheritance.

If education is to prepare learners not only to compute outcomes, but to grasp structures, causal relations, and spaces of possibility, then the challenge before us is less one of curricular innovation than of epistemic recalibration—an invitation to rethink what it means to know, to learn, and to reason in a world increasingly shaped by QIST and AI—domains in which understanding is inseparable from interaction, composition, and transformation.

This recalibration is opposite what contemporary schooling has privileged: abstract, decontextualized symbol manipulation as the primary marker of intellectual achievement. Such narrowing underutilizes core human cognitive capacities—evolutionarily we are equipped not only for symbolic reasoning, but for perceiving structure, aligning relations across representations, simulating dynamic processes, and constructing understanding through action. These capacities are precisely those required to engage with non-classical systems, integrate multiple representational modalities, and reason productively about complex, intelligent technologies.

Seen in this light, diagrammatic and process-oriented instructional innovations are not incremental innovations but signals of a deeper pedagogical shift—one that connects advanced scientific reasoning with forms of cognition that integrate theoretical insight, perceptual grounding, and constructive practice. The significance lies in improved accessibility, intuition, and their alignment with how understanding itself is formed—through the active organization of relations rather than the passive manipulation of symbols.

More broadly, we are witnessing the emergence of a new *cognitive economy*, in which human capital is dynamic and co-evolves with technological systems. The purpose of tech-driven pedagogy is more oriented toward cultivating adaptive, structurally informed thinkers—individuals capable of learning how to learn, of reasoning across representational forms, and of engaging creatively with systems that themselves evolve. The value of education can no longer be measured solely in years of schooling, but in the capacity for adaptation, innovation, and systemic thinking, which are most effectively cultivated through early exposure to cognitively rich and relevant frameworks. By introducing QPic, we create a corridor for such adaptive development.

## Data Availability

The original contributions presented in the study are included in the article/supplementary material, further inquiries can be directed to the corresponding author/s.

## Footnotes

1 For instance, in the summer of 2025, a study similar to the UK pilot study (Dündar-Coecke et al., 2023; Dündar-Coecke et al., 2025) was conducted in Pakistan. Two key differences were that the teaching was delivered in person rather than online, and the teaching was completed in 1 month instead of two. Nevertheless, the results were similar (Durrani, 2025). Another example is a qualitative study in Sweden that focused specifically on quantum teleportation (Kilde-Westberg et al., 2025).

## References

[B1] Abramsky S. Coecke B. (2004). “A categorical semantics of quantum protocols,” in Proceedings of the 19th Annual IEEE Symposium on Logic in Computer Science (LICS) (Los Alamitos, CA: IEEE Computer Society), 415–425. doi: 10.1109/LICS.2004.1319636

[B2] Abramsky S. Coecke B. (2009). Categorical quantum mechanics. Handb. Quant. Logic Quant. Struct. 2, 261–325. doi: 10.1016/B978-0-444-52869-8.50010-4

[B3] Aerts D. Coecke B. (1999). The Creation-Discovery-View: Towards a Possible Explanation of Quantum Reality. Dordrecht: Springer Netherlands. 105–116. doi: 10.1007/978-94-017-2043-4_11

[B4] Anderson L. W. Krathwohl D. R. (2001). A Taxonomy for Learning, Teaching, and Assessing: A Revision of Bloom's Taxonomy of Educational Objectives: Complete Edition. New York, NY: Addison Wesley Longman, Inc.

[B5] Autor D. (2022). The Labor Market Impacts of Technological Change: From Unbridled Enthusiasm to Qualified Optimism to Vast Uncertainty. Technical report. Cambridge, MA: National Bureau of Economic Research. doi: 10.3386/w30074

[B6] Aytaç K. (1980). Avrupa e&#*x0011F;itim tarihi: Antik ça*&#*x0011F;dan 19. yüzy*&#*x00131;l*&#*x00131;n sonlar*&#*x00131;na kadar*. Ankara: AÜ Dil ve Tarih-Companyğrafya Fak.

[B7] Backens M. Miller-Bakewell H. de Felice G. Lobski L. van de Wetering J. (2021). There and back again: a circuit extraction tale. Quantum 5:421. doi: 10.22331/q-2021-03-25-421

[B8] Backens M. Nabi Duman A. (2015). A complete graphical calculus for Spekkens' toy bit theory. Found. Phys. 46, 70–103. doi: 10.1007/s10701-015-9957-7

[B9] Becker G. S. (1918). Human Capital: A Theoretical and Empirical Analysis, with Special Reference to Education. Chicago, IL: University of Chicago Press.

[B10] Benacerraf P. (1973). Mathematical truth. J. Philos. 70, 661–679. doi: 10.2307/2025075

[B11] Bloom B. S. Engelhart M. D. Furst E. J. Hill W. H. Krathwohl D. R. (1956). Taxonomy of Educational Objectives: The Classification of Educational Goals. Handbook 1: Cognitive Domain. New York, NY: Longman.

[B12] Boisseau G. Sobociński P. (2021). String diagrammatic electrical circuit theory. arXiv Preprint arXiv:2106.07763. 372, 178–191. doi: 10.4204/EPTCS.372.13

[B13] Bombin H. Litinski D. Nickerson N. Pastawski F. Roberts S. (2024). Unifying flavors of fault tolerance with the zx calculus. Quantum 8:1379. doi: 10.22331/q-2024-06-18-1379

[B14] Bonchi F. Sobociński P. Zanasi F. (2021). “A survey of compositional signal flow theory,” in Advancing Research in Information and Communication Technology: IFIP's Exciting First 60+ Years, Views from the Technical Committees and Working Groups (Cham: Springer), 29–56. doi: 10.1007/978-3-030-81701-5_2

[B15] Bowles S. (1971). Unequal education and the reproduction of the social division of labor. Rev. Radic. Polit. Econ. 3, 1–30. doi: 10.1177/048661347100300401

[B16] Bowles S. Gintis H. (1976). Schooling in Capitalist America, Vol. 57. New York, NY: Basic Books.

[B17] Brouwer L. (1913). Intuitionism and formalism. Bull. Am. Math. Soc. 20, 81–96. doi: 10.1090/S0002-9904-1913-02440-6

[B18] Brynjolfsson E. McAfee A. (2014). The Second Machine Age: Work, Progress, and Prosperity in a Time of Brilliant Technologies. New York, NY: WW Norton and Company.

[B19] Butterworth B. (1999). A head for figures. Science 284, 928–929. doi: 10.1126/science.284.5416.928 10357680

[B20] Carette T. Horsman D. Perdrix S. (2019). Szx-Calculus: Scalable Graphical Quantum Reasoning, Vol. 138, 1–55. Schloss Dagstuhl – Leibniz-Zentrum f?r Informatik.

[B21] Carnap R. (1959). Logical Positivism. Glencoe, Il: Free Press.

[B22] Coecke B. (2006). “Kindergarten quantum mechanics: lecture notes,” in AIP Conference Proceedings, vol. 810 (New York, NY: American Institute of Physics), 81–98. doi: 10.1063/1.2158713

[B23] Coecke B. (2008). “Axiomatic description of mixed states from selinger's cpm-construction,” in Electronic Notes in Theoretical Computer Science, 210:3-13. Proceedings of the 4th International Workshop on Quantum Programming Languages (QPL 2006), Vol 210 (Amsterdam: Elsevier B.V.), 3–13. doi: 10.1016/j.entcs.2008.04.014

[B24] Coecke B. (2010). Quantum picturalism. Contemp. Phys. 51, Linguistic Analysis, 59–83. doi: 10.1080/00107510903257624

[B25] Coecke B. (2020). The Mathematics of Text Structure, Vol 20. Cham: Springer, 181–217. doi: 10.1007/978-3-030-66545-_6

[B26] Coecke B. de Felice G. Meichanetzidis K. Toumi A. (2020). Foundations for near-term quantum natural language processing. arXiv Preprint arXiv:2012.03755. doi: 10.48550/arXiv.2012.03755

[B27] Coecke B. Duncan R. (2008). “Interacting quantum observables,” in Proceedings of the 37th International Colloquium on Automata, Languages and Programming (ICALP), Lecture Notes in Computer Science, Vol. 5126 (Berlin, Heidelberg: Springer), 298–310. doi: 10.1007/978-3-540-70583-3_25

[B28] Coecke B. Duncan R. (2011). Interacting quantum observables: categorical algebra and diagrammatics. N. J. Phys. 13:043016. doi: 10.1088/1367-2630/13/4/043016

[B29] Coecke B. Edwards B. Spekkens R. W. (2011). Phase groups and the origin of non-locality for qubits. Electr. Notes Theoret. Comput. Sci. 270, 15–36. doi: 10.1016/j.entcs.2011.01.021

[B30] Coecke B. Kissinger A. (2017). Picturing Quantum Processes. A First Course in Quantum Theory and Diagrammatic Reasoning. Cambridge: Cambridge University Press. doi: 10.1017/9781316219317

[B31] Coecke B. Pavlović D. Vicary J. (2013). A new description of orthogonal bases. Math. Struct. Comput. Sci. 23, 555–567. doi: 10.1017/S0960129512000047

[B32] Coecke B. Sadrzadeh M. Clark S. (2010). “Mathematical foundations for a compositional distributional model of meaning,” in A Festschrift for Jim Lambek, volume 36 of Linguistic Analysis, eds. J. van Benthem, M. Moortgat, and W. and Buszkowski (Linguistic Analysis), 345–384.

[B33] de Beaudrap N. Bian X. Wang Q. (2020). Fast and effective techniques for T-count reduction via spider nest identities. arXiv Preprint arXiv:2004.05164. doi: 10.48550/arXiv.2004.05164

[B34] de Beaudrap N. Horsman D. (2020). The zx calculus is a language for surface code lattice surgery. Quantum 4:218. doi: 10.22331/q-2020-01-09-218

[B35] De Cruz H. De Smedt J. (2007). The role of intuitive ontologies in scientific understanding-the case of human evolution. Biol. Philos. 22, 351–368. doi: 10.1007/s10539-006-9036-8

[B36] De Cruz H. De Smedt J. (2013). Mathematical symbols as epistemic actions. Synthese 190, 3–19. doi: 10.1007/s11229-010-9837-9

[B37] de Felice G. Poór B. Comfort C. Yeh L. Kupper M. Cashman W. . (2026). A dataflow programming framework for linear optical distributed quantum computing. Quantum 10:1972. doi: 10.22331/q-2026-01-19-1972

[B38] de Felice G. Shaikh R. A. Poór B. Yeh L. Wang Q. Coecke B. (2023). Light-matter interaction in the zxw calculus. Electr. Proc. Theoret. Comp. Sci. 384, 20–46. doi: 10.4204/EPTCS.384.2

[B39] De Smedt B. Noël M.-P. Gilmore C. Ansari D. (2013). How do symbolic and non-symbolic numerical magnitude processing skills relate to individual differences in children's mathematical skills? A review of evidence from brain and behavior. Trends Neurosci. Educ. 2, 48–55. doi: 10.1016/j.tine.2013.06.001

[B40] Dehaene S. (2011). The Number Sense: How the Mind Creates Mathematics. New York, NY: OUP USA.

[B41] Donald M. (1991). Origins of the Modern Mind: Three Stages in the Evolution of Culture and Cognition. Cambridge, MA: Harvard University Press.

[B42] Donhauser A. Bitzenbauer P. Qerimi L. Heusler S. Küchemann S. Kuhn J. . (2024). Empirical insights into the effects of research-based teaching strategies in quantum education. Phys. Rev. Phys. Educ. Res. 20:020601. doi: 10.1103/PhysRevPhysEducRes.20.020601

[B43] Duncan R. Perdrix S. (2009). “Graph states and the necessity of Euler decomposition,” in Mathematical Theory and Computational Practice: 5th Conference on Computability in Europe, CiE 2009, Heidelberg, Germany, July 19-24, 2009. Proceedings 5 (Berlin: Springer), 167–177. doi: 10.1007/978-3-642-03073-4_18

[B44] Dündar-Coecke S. (2020). “Experts and students demand andragogical insights in learning beyond pedagogical,” in The European Conference on Education 2020: Official Conference Proceedings (Nagoya: IAFOR / The International Academic Forum), 141–156. doi: 10.22492/issn.2188-1162.2020.13

[B45] Dündar-Coecke S. Puca C. Yeh L. Waseem M. H. Pothos E. M. Cervoni T. . (2025). Making the quantum world accessible to young learners through quantum picturalism: an experimental study. arXiv Preprint arXiv:2504.01013.

[B46] Dündar-Coecke S. Yeh L. Puca C. Pfaendler S. M.-L. Waseem M. H. Cervoni T. . (2023). “Quantum picturalism: Learning quantum theory in high school,” in 2023 IEEE International Conference on Quantum Computing and Engineering (QCE), vol. 3 (New York, NY: IEEE), 21–32. doi: 10.1109/QCE57702.2023.20321

[B47] Duneau T. Bruhn S. Matos G. Laakkonen T. Santi K. Pearson A. . (2024). Scalable and interpretable quantum natural language processing: an implementation on trapped ions. arXiv Preprint arXiv:2409.08777. Available online at: https://arxiv.org/abs/2409.08777

[B48] Durrani M. (2025). From building a workforce to boosting research and education: future quantum leaders have their say. Physics World, 16 December 2025. Available online at: https://physicsworld.com/a/from-building-a-workforce-to-boosting-research-and-education-future-quantum-leaders-have-their-say/

[B49] East R. D. van de Wetering J. Chancellor N. Grushin A. G. (2022). Aklt-states as zx-diagrams: diagrammatic reasoning for quantum states. PRX Quant. 3:010302. doi: 10.1103/PRXQuantum.3.010302

[B50] Fischer G. Lundin J. Lindberg J. O. (2020). Rethinking and reinventing learning, education and collaboration in the digital age—from creating technologies to transforming cultures. Int. J. Inform. Learn. Technol. 37, 241–252. doi: 10.1108/IJILT-04-2020-0051

[B51] Gavranović B. (2024). Fundamental components of deep learning: a category-theoretic approach. arXiv Preprint arXiv:2403.13001. Available online at: https://arxiv.org/abs/2403.13001

[B52] Gentner D. (1983). Structure-mapping: a theoretical framework for analogy. Cogn. Sci. 7, 155–170. doi: 10.1016/S0364-0213(83)80009-3

[B53] German D.-A. Pias M. Xiang Q. Kuruvadi S. S. (2023). “A quantum abacus for teaching quantum algorithms,” in 2023 IEEE Frontiers in Education Conference (FIE) (New York, NY: IEEE), 1–9. doi: 10.1109/FIE58773.2023.10343217

[B54] Ghani N. Hedges J. Winschel V. Zahn P. (2018). “Compositional game theory,” in Proceedings of the 33rd Annual ACM/IEEE Symposium on Logic in Computer Science (New York, NY: ACM), 472–481. doi: 10.1145/3209108.3209165

[B55] Gidney C. Fowler A. G. (2019). Efficient magic state factories with a catalyzed |*CCZ*〉 to 2|*T*〉 transformation. Quantum 3:135. doi: 10.22331/q-2019-04-30-135

[B56] Gödel K. (1947). What is cantor's continuum problem? Am. Math. Monthly 54, 515–525. doi: 10.1080/00029890.1947.11991877

[B57] Heckman J. J. Kautz T. (2012). Hard evidence on soft skills. Lab. Econ. 19, 451–464. doi: 10.1016/j.labeco.2012.05.014 PMC3612993 23559694

[B58] Hegarty M. (2004). Mechanical reasoning by mental simulation. Trends Cogn. Sci. 8, 280–285. doi: 10.1016/j.tics.2004.04.001 15165554

[B59] Huang J. Li S. M. Yeh L. Kissinger A. Mosca M. Vasmer M. (2023). Graphical css code transformation using zx calculus. Electr. Proc. Theoret. Comput. Sci. 384, 1–19. doi: 10.4204/EPTCS.384.1

[B60] Johnston I. D. Crawford K. Fletcher P. R. (1998). Student difficulties in learning quantum mechanics. Int. J. Sci. Educ. 20, 427–446. doi: 10.1080/0950069980200404

[B61] Kahneman D. (2011). Thinking, fast and slow. macmillan.

[B62] Kilde-Westberg S. Johansson A. Pearson A. Enger J. (2025). Making sense of quantum teleportation: an intervention study on students' conceptions using a diagrammatic approach. arXiv Preprint arXiv:2511.21443. Available online at: https://arxiv.org/abs/2511.21443

[B63] Kissinger A. van de Wetering J. (2019a). Reducing T-count with the ZX-calculus. arXiv Preprint arXiv:1903.10477. Available online at: https://arxiv.org/abs/1903.10477

[B64] Kissinger A. van de Wetering J. (2019b). Universal mbqc with generalised parity-phase interactions and pauli measurements. Quantum 3:134. doi: 10.22331/q-2019-04-26-134

[B65] Kissinger A. van de Wetering J. (2025). Picturing Quantum Software. An Introduction to the ZX-Calculus and Quantum Compilation. Cambridge, MA: Cambridge University Press. Available online at: https://github.com/zxcalc/book

[B66] Knowles M. S. Associates (1984). Andragogy in Action: Applying Modern Principles of Adult Learning. Jossey-Basshigher education series. San Francisco, CA.

[B67] Koffka K. (1935). Principles of Gestalt Psychology. London: Routledge. doi: 10.4324/9781315009292

[B68] Kotseruba I. Tsotsos J. K. (2018). 40 years of cognitive architectures: core cognitive abilities and practical applications. Artif. Intell. Rev. 53, 17–94. doi: 10.1007/s10462-018-9646-y

[B69] Koziell-Pipe A. Yeung R. Sutcliffe M. (2024). “Towards faster quantum circuit simulation using graph decompositions, GNNs and reinforcement learning,” in The 4th Workshop on Mathematical Reasoning and AI at the 38th Conference on Neural Information Processing Systems (NeurIPS 2024) (NeurIPS MATH-AI Workshop).

[B70] Krijtenburg-Lewerissa K. Pol H. J. Brinkman A. van Joolingen W. R. (2017). Insights into teaching quantum mechanics in secondary and lower undergraduate education. Phys. Rev. Phys. Educ. Res. 13:010109. doi: 10.1103/PhysRevPhysEducRes.13.010109

[B71] Laakkonen T. Meichanetzidis K. Coecke B. (2024). Quantum algorithms for compositional text processing. Electron. Proc. Theor. Comput. Sci. 406, 162–196. doi: 10.4204/EPTCS.406.8

[B72] Lakoff G. Núñez R. (2000). Where Mathematics Comes from, Vol. 6. New York, NY: Basic Books.

[B73] Lorenz R. Pearson A. Meichanetzidis K. Kartsaklis D. Coecke B. (2023). Qnlp in practice: running compositional models of meaning on a quantum computer. J. Artif. Intell. Res. 76, 1305–1342. doi: 10.1613/jair.1.14329

[B74] Mau A. (2023). Barriers to Further Maths Summary Report. Technical report, Outreach Department. London: Imperial College London.

[B75] McDowall-Rose H. Shaikh R. A. Yeh L. (2025). From fermions to qubits: a zx-calculus perspective. arXiv Preprint arXiv:2505.06212.

[B76] McElvanney T. Backens M. (2023). Complete flow-preserving rewrite rules for mbqc patterns with pauli measurements. Electr. Proc. Theoret. Comput. Sci. 394, 66–82. doi: 10.4204/EPTCS.394.5

[B77] McKinsey Global Institute (2023). Generative AI and the Future of Work in America. New York, NY: McKinsey & Company. Available online at: https://www.mckinsey.com/mgi/our-research/generative-ai-and-the-future-of-work-in-america

[B78] Mock J. Huber S. Bloechle J. Dietrich J. F. Bahnmueller J. Rennig J. . (2018). Magnitude processing of symbolic and non-symbolic proportions: an fmri study. Behav. Brain Funct. 14:9. doi: 10.1186/s12993-018-0141-z 29747668 PMC5944011

[B79] Monti M. M. Parsons L. M. Osherson D. N. (2012). Thought beyond language: neural dissociation of algebra and natural language. Psychol. Sci. 23, 914–922. doi: 10.1177/0956797612437427 22760883

[B80] Núñez R. E. (2017). Is there really an evolved capacity for number? Trends Cogn. Sci. 21, 409–424. doi: 10.1016/j.tics.2017.03.005 28526128

[B81] OECD (2022). Micro-Credentials for Lifelong Learning and Employability: Uses and Possibilities. OECD Education Policy Perspectives, No. 66. Paris: OECD Publishing. doi: 10.1787/9c4b7b68-en

[B82] OECD (2024). Education at a Glance 2024: OECD Indicators. Paris: OECD Publishing. doi: 10.1787/c00cad36-en

[B83] Penrose R. (1971). “Applications of negative dimensional tensors,” in Combinatorial Mathematics and Its Applications, ed. D. J. A. Welsh (London: Academic Press), 221–244.

[B84] Pinzani N. Gogioso S. Coecke B. (2019). Categorical semantics for time travel. In 2019 34th Annual ACM/IEEE Symposium on Logic in Computer Science (LICS), pages 1-20. IEEE. doi: 10.1109/LICS.2019.8785664

[B85] Popper K. R. (1972). Objective Knowledge: An Evolutionary Approach. New York, NY: Oxford University Press.

[B86] Prize T. N. (2020). Nobel Prize in Physics 2020 – *Roger Penrose*–*facts*. Available online at: https://www.nobelprize.org/prizes/physics/2020/penrose/facts/ (Acessed January 03, 2026).

[B87] Psacharopoulos G. (1995). The Profitability of Investment in Education: Concepts and Methods, Vol. 63. Washington, DC: World Bank. Available online at:

[B88] Psacharopoulos G. Patrinos H. A. (2018). Returns to investment in education: a decennial review of the global literature. Educ. Econ. 26, 445–458. doi: 10.1080/09645292.2018.1484426

[B89] Richardson J. Bourdieu P. (1986). The forms of capital. Handb. Theory Res. Sociol. Educ. 241:258.

[B90] Rodatz B. Poór B. Kissinger A. (2024). Floquetifying stabiliser codes with distance-preserving rewrites. arXiv Preprint arXiv:2410.17240. Available online at: https://arxiv.org/abs/2410.17240

[B91] Sadrzadeh M. (2017). Quantization, frobenius and bi algebras from the categorical framework of quantum mechanics to natural language semantics. Front. Phys. 5:18. doi: 10.3389/fphy.2017.00018

[B92] Scottish Qualifications Authority (2019). Advanced Higher Mathematics Course Specification. Glasgow: Scottish Qualifications Authority.

[B93] Scottish Qualifications Authority (2023). 2023 Advanced Higher Mathematics Course Report. Technical Report. Glasgow: Scottish Qualifications Authority.

[B94] Scottish Qualifications Authority (2024). Provisional Attainment Statistics - *August 2024*. Glasgow: Scottish Qualifications Authority.

[B95] Selinger P. (2007). Dagger compact closed categories and completely positive maps. Electron. Notes Theor. Comput. Sci. 170, 139–163. doi: 10.1016/j.entcs.2006.12.018

[B96] Sfard A. (1991). On the dual nature of mathematical conceptions: reflections on processes and objects as different sides of the same coin. Educ. Stud. Math. 22, 1–36. doi: 10.1007/BF00302715

[B97] Shaikh R. A. Wang Q. Yeung R. (2023). How to sum and exponentiate hamiltonians in zxw calculus. Electr. Proc. Theoret. Comput. Sci. 394, 236–261. doi: 10.4204/EPTCS.394.14

[B98] Shaikh R. A. Yeh L. Gogioso S. (2024). The focked-up zx calculus: picturing continuous-variable quantum computation. arXiv Preprint arXiv:2406.02905.

[B99] Shapiro S. (2000). Thinking About Mathematics: The Philosophy of Mathematics. Oxford: OUP Oxford. doi: 10.1093/0195139305.001.0001

[B100] Sieg W. (2013). Hilbert's Programs and Beyond. New York, NY: Oxford University Press.

[B101] Singh C. (2008). Student understanding of quantum mechanics at the beginning of graduate instruction. Am. J. Phys. 76, 277–287. doi: 10.1119/1.2825387

[B102] Slotnick S. D. Thompson W. L. Kosslyn S. M. (2005). Visual mental imagery induces retinotopically organized activation of early visual areas. Cereb. Cortex 15, 1570–1583. doi: 10.1093/cercor/bhi035 15689519

[B103] The Office of Qualifications and Examinations Regulation (2016). GCE Subject Level Conditions and Requirements for Mathematics. Coventry: The Office of Qualifications and Examinations Regulation.

[B104] Tolmie A. Dündar-Coecke S. (2020). “Lifespan conceptual development in science: brain and behavior,” in Educational Neuroscience: Development Across the Life Span, eds. M. S. C. Thomas, D. Mareschal, and I. Dumontheil (New York, NY: Routledge), 193–220. doi: 10.4324/9781003016830-11

[B105] Townsend-Teague A. Magdalena de la Fuente J. Kesselring M. (2023). “Floquetifying the colour code,” in Proceedings of the Twentieth International Conference on Quantum Physics and Logic, Paris, France, 17-21st July 2023, volume 384 of Electronic Proceedings in Theoretical Computer Science, eds. S. Mansfield, B. Valîron, and V. Zamdzhiev (Waterloo: Open Publishing Association), 265–303. doi: 10.4204/EPTCS.384.14

[B106] Treisman A. M. Gelade G. (1980). A feature-integration theory of attention. Cogn. Psychol. 12, 97–136. doi: 10.1016/0010-0285(80)90005-5 7351125

[B107] Tull S. Lorenz R. Clark S. Khan I. Coecke B. (2024). Towards Compositional Interpretability for Xai. arXiv.

[B108] Tversky B. (2013). “Visualizing thought,” in Handbook of Human Centric Visualization (New York, NY: Springer), 3–40.

[B109] Tversky B. (2019). Mind in Motion: How Action Shapes Thought. New York, NY: Basic Books. doi: 10.1145/3325480.3325525

[B110] Varela F. J. Thompson E. Rosch E. (2016). The Embodied Mind: Cognitive Science and Human Experience, Revised edition. Cambridge, MA: MIT Press.

[B111] Wang Q. East R. D. P. Shaikh R. A. Yeh L. Poór B. Coecke B. (2025). Beyond Penrose tensor diagrams with the ZX calculus: applications to quantum computing, quantum machine learning, condensed matter physics, and quantum gravity. arXiv Preprint arXiv: 2511.06012. Available online at: https://arxiv.org/abs/2511.06012

[B112] Wertheimer M. (1923). Laws of organization in perceptual forms. Psycologische Forschung 4, 301–350.

[B113] Whitehead A. N. Russell B. (1910–1913). Principia mathematica, 1st Edn. 3 volumes published 1910 (Vol. I), 1912 (Vol. II), 1913 (Vol. III). Cambridge: Cambridge University Press.

[B114] World Economic Forum (2023). The future of jobs report 2023. World Economic Forum. Available online at: https://www.weforum.org/publications/the-future-of-jobs-report-2023/

[B115] Yeh L. (2026). Transdimensional Quantum Computation: The Graphical Novel (Ph.D. thesis). Oxford: University of Oxford.

